# High Guanidinium Permeability Reveals Dehydration-Dependent Ion Selectivity in the Plasmodial Surface Anion Channel

**DOI:** 10.1155/2014/741024

**Published:** 2014-08-27

**Authors:** Abdullah A. B. Bokhari, Neida K. Mita-Mendoza, Alexandra Fuller, Ajay D. Pillai, Sanjay A. Desai

**Affiliations:** The Laboratory of Malaria and Vector Research, National Institute of Allergy and Infectious Diseases, National Institutes of Health, Rockville, MD 20852, USA

## Abstract

Malaria parasites grow within vertebrate erythrocytes and increase host cell permeability to access nutrients from plasma. This increase is mediated by the plasmodial surface anion channel (PSAC), an unusual ion channel linked to the conserved *clag* gene family. Although PSAC recognizes and transports a broad range of uncharged and charged solutes, it must efficiently exclude the small Na^+^ ion to maintain infected cell osmotic stability. Here, we examine possible mechanisms for this remarkable solute selectivity. We identify guanidinium as an organic cation with high permeability into human erythrocytes infected with *Plasmodium falciparum*, but negligible uptake by uninfected cells. Transport characteristics and pharmacology indicate that this uptake is specifically mediated by PSAC. The rank order of organic and inorganic cation permeabilities suggests cation dehydration as the rate-limiting step in transport through the channel. The high guanidinium permeability of infected cells also allows rapid and stringent synchronization of parasite cultures, as required for molecular and cellular studies of this pathogen. These studies provide important insights into how nutrients and ions are transported via PSAC, an established target for antimalarial drug development.

## 1. Introduction

Malaria parasites are intracellular pathogens that invade, grow, and replicate asexually within erythrocytes; the clinical sequelae of malaria are, in large measure, determined by modification and eventual destruction of host erythrocytes. During its ~48 h intracellular cycle, the human pathogen* P. falciparum* remodels its host cell by generating a membranous network in erythrocyte cytosol and altering erythrocyte membrane properties such as adhesiveness and permeability to various organic and inorganic solutes [1–4].

This increased permeability is mediated by the plasmodial surface anion channel (PSAC), identified by patch-clamp studies of the host cell membrane [5]. PSAC activity and the associated* clag* multigene family are conserved in all* Plasmodium* spp. [6–9], suggesting a function required for intracellular parasite survival.* In vitro* growth inhibition studies using PSAC inhibitors and modified media have implicated an essential role in parasite nutrient acquisition [10], with sugars, amino acids, purines, and some vitamins all having established uptake [11–13].

As a shared ion channel for these structurally divergent nutrients, PSAC has broad selectivity for solutes of varying charge and size [11, 14, 15]. At the same time, this channel must efficiently exclude Na^+^, an impermeant cation responsible for the osmotic stability of erythrocytes in plasma [16]. PSAC excludes Na^+^ by 10^3.5^- to 10^5^-fold when compared to Cl^−^, a remarkable feat for a channel that allows large organic cations to pass [14]. Consistent with fine-tuning of PSAC solute selectivity by evolutionary pressures, this level of Na^+^ exclusion is sufficient to prevent osmotic lysis of infected cells before completion of the parasite's intracellular cycle [17]. Although being low, Na^+^ flux through this channel is sufficient to remodel the host erythrocyte's cation concentrations and affect parasite activities [17–19]. Structure-function studies with mammalian ion channels have revealed mechanisms for selecting a specific ion and excluding nearly all other solutes [20], but the reverse problem—broad permeability with exclusion of the small Na^+^ ion by this parasite channel—requires distinct solutions that will have fundamental implications for understanding permeation.

Here, we examine possible mechanisms for PSAC's unusual selectivity and identify guanidinium (Gdm^+^) as a cation with high permeability. We show that monovalent cations have permeabilities that increase with cation ionic radius, contradicting pore sieving predictions and paralleling a similar relationship for anions in this channel. High Gdm^+^ permeability also enables stringent synchronization of parasite cultures, as commonly needed for molecular and cellular studies of malaria parasites. These findings suggest a testable model for how the channel achieves its unusual solute selectivity.

## 2. Materials and Methods

### 2.1. Parasite Cultivation and Synchronization

Human erythrocytes were obtained from anonymous donors (Interstate Blood Bank, Memphis, TN) and used for* in vitro P.  falciparum* cultivation of indicated parasite lines in RPMI-1640 medium supplemented with 0.5% lipid-rich bovine albumin (MP Biomedicals, Solon, OH); cultures were maintained at 37°C under 5% O_2_, 5% CO_2_, and 90% N_2_.

To assess the efficiency of synchronization conditions, asynchronous parasite cultures were treated with either 300 mM D-sorbitol or 150 mM guanidinium chloride (Gdm-Cl) in a buffered solution (20 mM HEPES, 0.1 mg/mL BSA, pH 7.4 with NaOH); each experiment included treatment with standard culture medium as a matched control. Synchronization involved 5 or 30 min incubations at room temperature and was terminated by addition of 10 volumes of culture medium. After centrifugation to remove the lysis solution, the cells were resuspended in culture medium and returned to 37°C for cultivation without additional washing. Parasite stage and growth were evaluated after 24 h using microscopic examination of Giemsa-stained smears.

### 2.2. Osmotic Lysis Transport Assays

Solute transport assays were performed as described previously [21]. Trophozoite-infected erythrocytes were harvested and enriched using the percoll/sorbitol method, washed, and resuspended at 0.1% hematocrit in osmotic lysis solutions containing either 280 mM sorbitol or 150 mM Gdm-Cl buffered with 20 mM HEPES, 0.1 mg/mL BSA, pH 7.4. The permeability of other cations was identically measured; each salt produced negligible hemolysis of uninfected cells (not shown). Where present, inhibitors were added from DMSO stock solutions. Solute transport was quantified by tracking transmittance of 700 nm light through a 1 mL cell suspension; kinetics were measured at indicated temperatures using a spectrophotometer (DU640 with Peltier temperature control, Beckman Coulter, Fullerton, CA). Inhibitor dose response experiments were normalized to matched controls without inhibitor; a normalized permeability at each inhibitor concentration (*P*_*i*_) was calculated according to *P*_*i*_ = *t*_*o*_/*t*_*i*_, where *t*_*o*_ and *t*_*i*_ correspond to the time required to reach a threshold level of lysis without and with inhibitor, respectively. This equation is based on a quantitative and inverse relationship between solute transport and time to cell lysis [21]. Permeability estimates and inhibitor affinities determined using this method match those obtained with tracer flux and patch-clamp [5, 7, 10, 21].

### 2.3. Sybr Green Measurements

Toxicity of Gdm-Cl was evaluated using parasite cultures after synchronization with two consecutive 30 min D-sorbitol treatments. These synchronized cultures were treated with either 150 mM Gdm-Cl, 20 mM HEPES, 0.1 mg/mL BSA, pH 7.4, or culture medium for 5 min at room temperature. After adding 10 volumes of culture medium, the cells were centrifuged to remove the medium and resuspended to 2% hematocrit in culture medium prior to plating in 96-well microplates. After cultivation for 72 h, parasite DNA production was quantified with SYBR Green I nucleic acid stain as described previously [17].

## 3. Results

### 3.1. High PSAC Permeability to Guanidinium^+^

**Figure 1:**
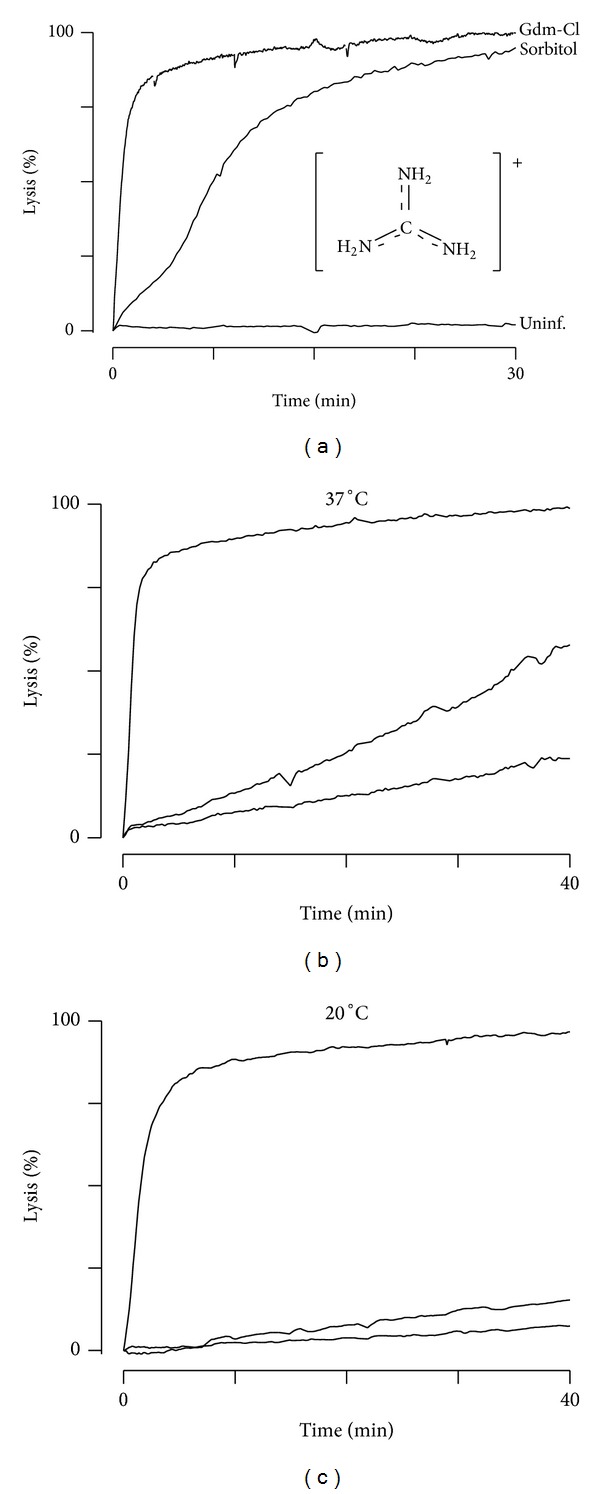
High Gdm^+^ permeability in infected but not uninfected RBCs. (a) Osmotic lysis kinetics for infected erythrocytes in Gdm-Cl or sorbitol at 37°C (top two traces). Notice the faster kinetics in Gdm-Cl. Uninfected cells do not lyse in Gdm-Cl (bottom trace). Inset shows the structure of Gdm^+^, which has a net +1 charge distributed amongst three primary amines. (b and c) Osmotic lysis kinetics for infected cells in Gdm-Cl with 0, 200, or 2000 *μ*M furosemide (top to bottom traces, resp.). 200 *μ*M furosemide produces incomplete inhibition at 37°C but is more effective at 20°C (panels b and c, resp.), suggesting that Gdm^+^ is an R+ solute.

We sought to examine PSAC's unusual solute selectivity profile and recognized that blasticidin S and leupeptin, toxins that reach their intracellular parasite targets via PSAC [22–24], are both bulky guanidine derivatives with molecular weights >420 Da. Unsubstituted guanidine is positively charged at physiological pH and its conjugate acid, the guanidinium ion (Gdm^+^, Figure 1(a) inset), has been used to study transport through other ion channels [25]. We therefore examined Gdm^+^ permeability in infected erythrocytes and used a quantitative transmittance assay [26]. These studies revealed rapid osmotic lysis of infected cells in isotonic Gdm-Cl; the half-time, 0.89 ± 0.08 min, was significantly less than in isotonic sorbitol (6.7 ± 0.5 min, *P* < 10^−8^), a highly permeant sugar alcohol. Although net uptake of the Gdm-Cl salt depends on both Gdm^+^ and Cl^−^ permeabilities to maintain electroneutrality, conductive Cl^−^ transport at this membrane is greater and not rate-limiting [4, 21]. Because there is a quantitative and inverse relationship between osmotic lysis half-time and solute permeability [21], these measurements implicate 8-fold greater permeability for Gdm^+^ than for sorbitol. In contrast, uninfected human erythrocytes exhibited low Gdm^+^ permeability and were osmotically stable in Gdm-Cl (bottom trace, Figure 1(a)).

The nonspecific PSAC inhibitor, furosemide, inhibited Gdm^+^ uptake, suggesting channel-mediated transport (Figure 1(b)) [27]. Notably, the levels of inhibition achieved with 200 *μ*M and 2 mM concentrations of furosemide resembled those for a subset of PSAC substrates that access two different mechanisms of transport through this channel [15, 28]. These two mechanisms exhibit differences in inhibitor efficacy: while the transport of some solutes is abolished by 200 *μ*M furosemide, other solutes, collectively referred to as “R+” solutes, exhibit significant residual uptake via PSAC that can be blocked by a higher furosemide concentration (2 mM). Remarkably, PSAC inhibitors from multiple chemical scaffolds exhibit a similar 10-fold reduction in potency when transport is examined with each R+ solute. This observation suggests two distinct mechanisms used by this channel to recognize and transport solutes. Because the residual transport mechanism has steep temperature dependence [28], we examined Gdm^+^ transport at 20°C; under this condition, 200 *μ*M furosemide largely abolished uptake (Figure 1(c)), as reported for all other known R+ solutes [15]. These experiments suggest Gdm^+^ is transported via PSAC as an R+ solute.

Because furosemide is nonspecific, we examined the mechanism of Gdm^+^ uptake further with ISPA-28, a potent and specific small molecule inhibitor identified by high-throughput screening [7]. ISPA-28 blocks PSAC activity associated with the Dd2 parasite line (*K*_0.5_ = 56 nM) but is largely inactive against channel activity induced by other parasite lines such as HB3 (*K*_0.5_ = 43 *μ*M); this compound's unique specificity enabled identification of* clag3* genes and the channel's role in nutrient uptake through genetic mapping and DNA transfection experiments [7, 10]. A short variable motif on the CLAG3 protein is exposed at the host cell surface and has been implicated in ISPA-28 binding [29].

**Figure 2:**
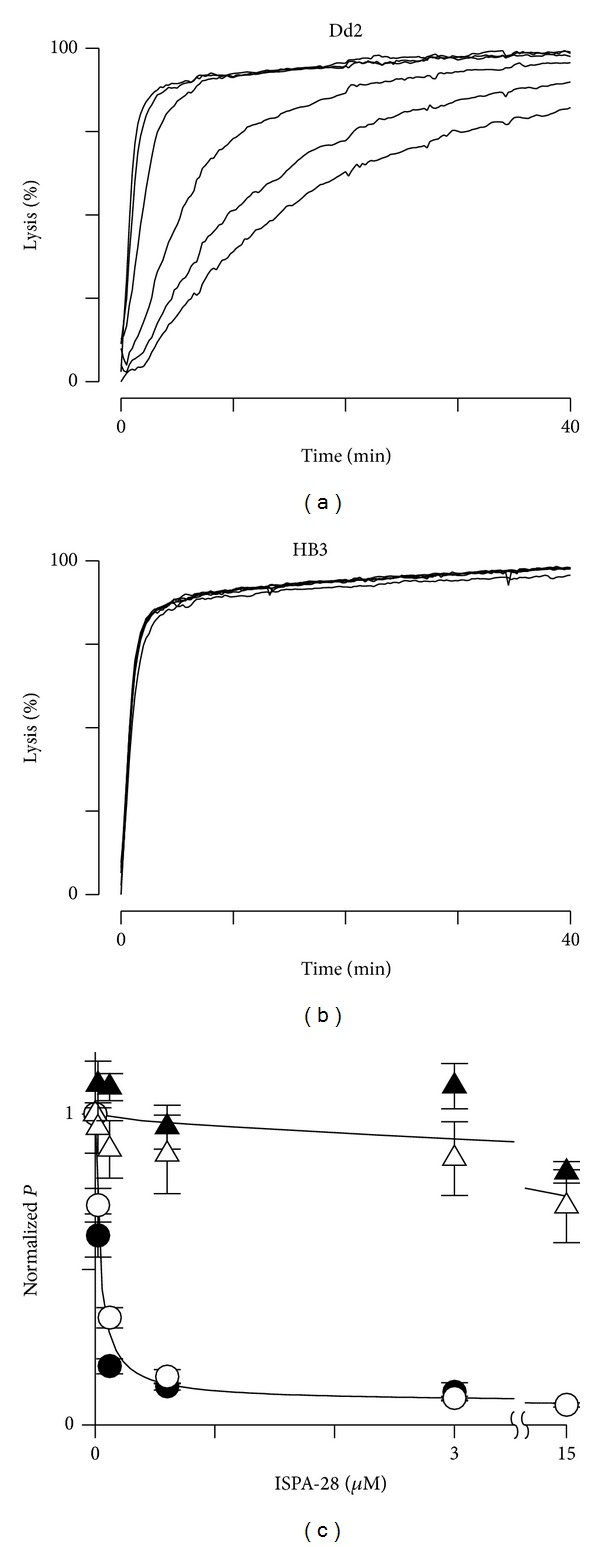
Gdm^+^ uptake is primarily via PSAC. (a and b) Osmotic lysis kinetics for Gdm^+^ uptake into Dd2 and HB3 at 15°C. Traces reflect inhibition dose responses using 0, 0.024, 0.12, 0.6, 3.0, and 15 *μ*M ISPA-28 (top to bottom, resp., in each panel). While inhibition is clear with Dd2-infected cells, there is negligible effect with HB3-infected cells. (c) Symbols represent mean ± S.E.M. of tallied dose responses from experiments as in panels (a) and (b) for Gdm-Cl and sorbitol (white and black symbols, resp.) using Dd2- and HB3-infected cells (circles and triangles, resp.). The Gdm^+^ and sorbitol dose responses do not differ (*n* = 3 trials at each concentration, *P* > 0.1 for comparisons between solutes in each parasite.).

ISPA-28 inhibited Gdm^+^ uptake into cells infected with Dd2 but not those infected with HB3 parasites (Figures 2(a) and 2(b)). To explore whether other transporters contribute to Gdm^+^ uptake after infection, we quantified ISPA-28 inhibition and compared block to that for sorbitol, a solute whose uptake via PSAC is well-established [7, 26]; these transport inhibition studies were performed at 15°C to reduce errors due to the residual transport mechanism described above. In both Dd2 and HB3 parasite lines, these dose response studies revealed quantitatively concordant inhibition of Gdm^+^ and sorbitol uptake by ISPA-28 (Figure 2(b)), indicating that Gdm^+^ uptake is mediated primarily by PSAC.

### 3.2. Low Toxicity of Gdm^+^ Permits Stringent Synchronization of Parasite Cultures

Sorbitol treatment, the current method of choice for synchronizing parasite cultures [30], is based on osmotic lysis of trophozoite-infected cells due to PSAC-mediated uptake [21]; it spares immature ring-infected cells, which lack this channel activity [31]. Two limitations include a requirement for relatively long incubations in sorbitol and poor stringency of synchronization. To achieve tighter synchrony for molecular studies such as stage-specific gene transcription, it is often necessary to use two or more rounds of sorbitol synchronization, making the procedure time- and effort-intensive. Alternative methods, such as gelatin floatation or enrichment of mature infected cells on magnetic columns [32, 33], have low yield or are also time-consuming.

**Figure 3:**
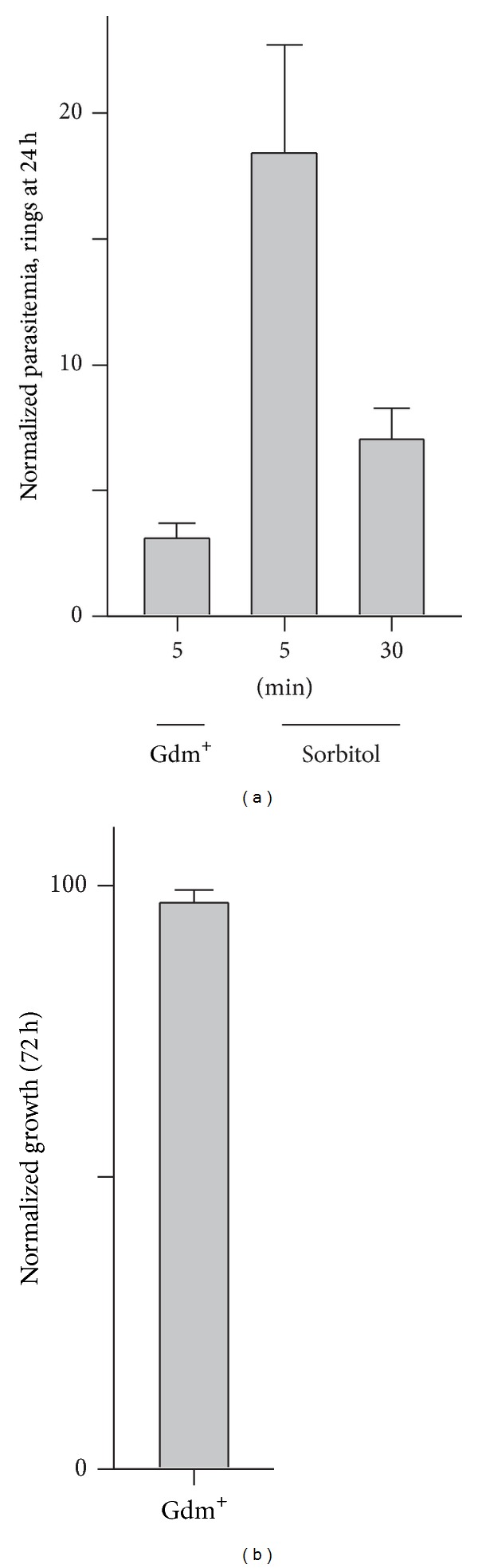
Stringent synchronization of cultures using Gdm-Cl. (a) Mature infected cells surviving synchronization with Gdm-Cl or sorbitol for indicated durations, as quantified using ring-stage parasitemia 24 h after treatment. A 5 min Gdm-Cl treatment is the most effective. (b) 72 h parasite growth after a 5 min treatment of synchronous cultures with Gdm-Cl, normalized to matched cultures sham-treated with culture medium. Bars represent mean ± S.E.M. of 9 replicates from 3 experiments.

We tested whether the greater permeability of Gdm^+^ allows improved synchronization by treating asynchronous cultures with either Gdm-Cl or sorbitol. A 5 min Gdm-Cl treatment was more effective than either a 5 or 30 min sorbitol exposure, as quantified with examination of Giemsa-stained smears after subsequent cultivation for 24 h (Figure 3(a), *P* < 0.05). Although improved synchronization is presumably determined by the greater Gdm^+^ permeability, the near-physiological ionic strength of the Gdm-Cl solution may also help; the lower ionic strength of sorbitol solutions may cause erythrocyte aggregation [34], leading to delayed solute uptake and osmotic lysis of some cells.

Because Gdm^+^ is a strong protein denaturant at high concentrations, we wondered whether this synchronization strategy would be toxic to parasite cultures. We therefore treated ring-stage cultures with isotonic Gdm-Cl solution and quantified subsequent parasite growth. Comparison to a sham treatment using standard culture medium revealed no change in parasite growth (Figure 3(b), *P* = 0.36), indicating that this treatment is not toxic to cultures. Experiments using a significantly longer Gdm^+^ treatment of 30 min yielded measurable toxicity, but we did not detect accumulated toxicity with prolonged, regular use of 5 min Gdm-Cl treatments over consecutive asexual cycles (not shown).

Protein denaturation by Gdm-Cl also cannot account for the apparent high Gdm^+^ permeability via PSAC. Such models would predict hemolysis of uninfected erythrocytes in Gdm-Cl solutions, which was not detected (Figure 1(a)). Denaturation would also not be consistent with block by ISPA-28, a highly specific PSAC inhibitor. Quantitatively concordant dose responses for inhibition of Gdm^+^ and sorbitol uptake (Figure 2), when combined with insights from single channel patch-clamp using this inhibitor [7], implicate permeation through a channel pore not compromised by Gdm-Cl exposure.

### 3.3. PSAC Permeabilities to Other Monovalent Cations

**Figure 4:**
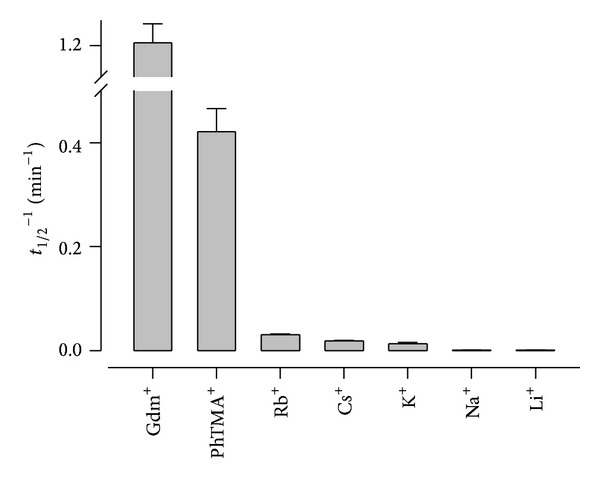
Dehydration-dependent permeation of cations. Mean ± S.E.M. apparent permeability coefficients for indicated cations, determined as the reciprocal of the osmotic lysis halftime in buffered solutions of each chloride salt at 37°C.

In contrast to Gdm^+^ and various organic cations [14, 35], PSAC maintains a very low Na^+^ permeability [16]. To explore possible mechanisms, we quantified the relative permeabilities of organic and inorganic cations with osmotic lysis kinetics for infected cells in buffered solutions of each chloride salt (Figure 4). As expected, Gdm^+^ was the most permeant of these cations. PhTMA^+^ had substantial permeability that was 3-fold lower. Cs^+^, Rb^+^, and K^+^, large group 1A alkali metals with ionic radii of 1.67, 1.48, and 1.33 Ǻ, respectively, had more modest but still clearly resolved uptake. Na^+^ and Li^+^, smaller group 1A metals with radii of 0.98 and 0.68 Ǻ, respectively, had negligible permeabilities. While anion fluxes through PSAC have been studied using both single-channel and whole-cell patch-clamp configurations, the significantly lower permeabilities of the cations in Figure 4 prohibit measurement of cation-specific currents with patch-clamp methods. These currents would be overwhelmed by the larger fluxes of anions such as Cl^−^, necessarily present at stoichiometric levels due to electroneutrality.

## 4. Discussion

The increased permeability of infected erythrocytes to small solutes is one of the earliest identified cellular phenotypes in malaria research [36]; studies from many groups have defined the range of permeant solutes and identified inhibitors [3, 14, 37–39]. Although several distinct channels have been proposed for the infected cell membrane [40], recent chemical screens and molecular studies have implicated PSAC as a shared route for most solutes with increased permeability [7, 26]. Parasite CLAG proteins, which lack homology to known channel proteins from other organisms, play a critical but incompletely understood role in formation of this channel [8, 10]. Permeating solutes may be uncharged or zwitterionic or may carry a net positive or negative charge. Solutes up to 670 Da in size have significant uptake [16], but the small Na^+^ and Li^+^ ions are effectively excluded (Figure 4). The combination of broad permeability to large solutes and effective exclusion of specific small ions is without precedent amongst other ion channels. This unusual selectivity profile appears to have been selected by evolutionary pressures that require uptake of diverse nutritive solutes, evasion of host immune responses, and a very low Na^+^ permeability to prevent osmotic lysis of infected cells in the bloodstream, where Na^+^ is the main osmotically active solute [41].

Here, we examined this unprecedented solute selectivity by quantifying cation transport through PSAC. We found that permeability increased with ionic size for group 1A cations (Figure 4), paralleling a similar relationship described for halide and pseudohalide anions identified through patch-clamp, SCN^−^ ≫ I^−^ > Br^−^ > Cl^−^ [4]. These findings contradict the predictions of simple pore sieving models, which expect the smallest solutes to have the greatest permeabilities. Instead, there appears to be a controlling effect of ion dehydration, the process of removing the shell of water molecules around dissolved ions [42, 43]. For both cation and anion series, greater PSAC permeability correlates precisely with lower energy requirement for dehydration.

In particular, Gdm^+^ and SCN^−^ are notable as the cation and anion with the fastest transport rates. Gdm^+^ is one of the most weakly hydrated ions known; it interacts poorly with water because a single positive charge is diffusely shared by three nitrogen atoms and because it has a rigid structure unable to interact well with water molecules [44]. SCN^−^ also has a low charge density and is the least hydrated of all the anions in the Hofmeister series [45]. The poor hydration of these ions also accounts for their strong denaturant properties when present at higher concentrations [44].

Studies on K^+^, Na^+^, and Ca^++^ channels suggest that permeating ions must be dehydrated to fit within the pore [20, 46]; dehydration is thought to allow specific interactions with the channel protein and enable selective transport. In this context, it is surprising that our studies implicate dehydration as a critical step in transport through PSAC: broad permeability to bulky organic solutes typically suggests a large pore capable of accommodating hydrated ions. We propose that ion dehydration may serve a distinct role in this channel's case by facilitating the selective exclusion of Na^+^. The energy required to dehydrate Na^+^, 91.2 kcal/mol [46], is very large indeed. Na^+^ channels compensate for this energy barrier by providing a strong binding site for Na^+^ in the pore; in contrast, the PSAC pore offers negligible compensation, with an Eisenman selectivity sequence that corresponds to the weakest theoretical binding site for permeating ions. Under such conditions, large, easily dehydrated ions and nutritive solutes will be preferred; Na^+^ and Li^+^ will be effectively excluded. Two observations implicate additional unknown factors in defining PSAC solute selectivity. First, it is not clear how small ions with intact water shells are excluded by a pore large enough to accommodate bulky organic solutes. Second, studies have found important differences in the transport of closely related organic solutes [15].

Our study also provides an improved, shorter protocol for synchronization of parasite cultures, as often required in basic research studies of transcription or translation stage-specificity. It may also be useful for parasite lines that do not tolerate extended exposure to ambient temperature and O_2_ levels, as are invariably associated with longer protocols.

Although identification of parasite* clag* genes as determinants of PSAC activity addressed long-standing debates about whether the channel is host- or pathogen-derived [7, 9, 40], the structural basis of solute recognition, binding, and transport through this channel remains largely unknown. The CLAG proteins lack conventional transmembrane domains for pore formation; they have also been proposed to serve unrelated roles in erythrocyte invasion or cytoadherence [47, 48]. Functional studies, such as those presented here, should guide inquiries into the molecular and structural basis of permeation through this unusual channel and important antimalarial drug target.

## Abbreviations

PSAC: Plasmodial surface anion channel
Gdm^+^: Guanidinium
Gdm-Cl: Guanidinium chloride.
